# Evaluating renal microcirculation in patients with acute kidney injury by contrast-enhanced ultrasonography: a protocol for an observational cohort study

**DOI:** 10.1186/s12882-022-03021-0

**Published:** 2022-12-08

**Authors:** Xiangyu Wang, Luzeng Chen, Tao Su

**Affiliations:** 1 Department of Ultrasound, Peking University First Hospital, Beijing, China; 2 Renal Division, Department of Medicine, Peking University First Hospital, Beijing, China; 3 Institute of Nephrology, Peking University, No 8, Xishiku Street, Xicheng District, Beijing, 100034 China

**Keywords:** Acute kidney injury, Microcirculation, Contrast-enhanced ultrasonography, Tubulointerstitial nephritis, Diagnosis and prognosis

## Abstract

**Background:**

Acute kidney injury (AKI) in critically ill patients has poor renal outcome with high mortality. Changes in intra-renal microcirculation and tissue oxygenation are currently considered essential pathophysiological mechanisms to the development and progression of AKI. This study aims to investigate the characteristics of contrast-enhanced ultrasonography (CEUS) derived parameters in biopsy-proven AKI patients, and examine the predictive value of these markers for renal outcome.

**Methods and design:**

This prospective observational study will enroll AKI patients who are diagnosed and staging following KDIGO (Kidney Disease: Improving Global Outcomes) criteria. All patients undergo a kidney biopsy and pathological tubulointerstitial nephropathy is confirmed. The CEUS examination will be performed at 0, 4 and 12 weeks after biopsy to monitor renal microcirculation. The percentage decrease of serum creatinine, 4-week and 12-week eGFR (estimated glomerular filtration rate) will also be reviewed as renal prognosis. The relationship of CEUS parameters with clinical and pathological markers will be analyzed. We perform a lassologit procedure to select potential affecting variables, including clinical, laboratory indexes and CEUS markers, to be included in the logistic regression model, and examine their predictive performance to AKI outcomes.

**Discussion:**

If we are able to show that CEUS derived parameters contribute to diagnosis and prognosis of AKI, the quality of life of patients will be improved while healthcare costs will be reduced.

**Trial registration:**

This study is retrospectively registered on the Chinese Medical Research Registration information System(https://61.49.19.26/login) on December 31, 2021: MR-11–22-003,503. This study has been approved by the Ethics and Scientific Research Department of Peking University First Hospital.

## Background

Acute kidney injury (AKI) is a syndrome characterized by a rapid decline in kidney function within a few hours to a few days [1]. The prognosis of AKI is poor, and it has been reported that the mortality rate of AKI in critically ill patients can even reach 80 percent in some studies [2]. Kidney damage varies according to the primary insult, including kidney ischemia, nephrotoxins exposure, dehydration or sepsis. Changes in intra-renal microcirculation and tissue oxygenation are currently considered essential pathophysiological mechanisms contributing to the development and progression of AKI [3–6]. Early morphological alterations are driven by a delicate balance between energy demand and oxygen supply [7]. Functional changes in renal microcirculation are common initial factors, that timely fluid and vasoactive agents therapies could make AKI reversible potentially. Therefore, it is fundamental to identify the decrease of intra-renal micro-perfusion early.

Common clinical imaging examinations include computed tomography (CT), magnetic resonance imaging (MRI), and positron emission tomography, which cannot reflect renal microperfusion. It is necessary to avoid contrast-induced kidney injury by enhanced CT or MR imaging for prevention of AKI occurrence and progression. Contrast-enhanced ultrasonography (CEUS) is a technique that enables reflecting the kidney perfusion quantification at the bedside for critically ill patients sensitively [8]. The main output parameters are relative blood volume (rBV), rising time (RT), mean transit time (mTT), peak intensity (PI), and cortex area under receiver operating characteristics curve (AUC) et al. Previous studies on chronic kidney disease (CKD) and transplanted kidneys highlighted the role of CEUS in evaluating parenchymal micro-vascularization and pathology. Patients in the advanced stage of the DKD had decreased renal cortex AUC and consistent change of microperfusion [9–11]. A lower PI and a higher mTT was found in early septic AKI indicating the occurrence of decreased cortical renal perfusion. mTT was significantly higher in patients with severe AKI [5], however the PI was not different from those with no AKI over the initial 3 days. In an ischemia–reperfusion injury mice model [12], 45-min IRI AKI caused severe as well as persistent renal perfusion impairment than that of 20-min IRI AKI. The decreased AUC at days 1–21, which representing lower renal cortical perfusion, could predict interstitial fibrosis 42 days after AKI. It was also reported that acute decompensated heart failure patients who subsequently developed AKI exhibited a significant lower renal cortical perfusion on admission (48 ± 8 dB/s) compared with healthy controls (60 ± 10 dB/s) and those who had ADHF(acute decompensated heart failure) but did not develop AKI (57 ± 9 dB/s). These results indicate that CEUS enables the evaluation of renal perfusion impairment, and associated with CKD after ischemic AKI and may serve as a noninvasive technique for assessing AKI-CKD progression. Another reason for recommendation of CEUS especially to patients with AKI is that ultrasonic contrast has no nephrotoxicity, the lung mainly discharges contrast agents. However, evidence based on the process of quantitative monitoring of intra-renal microcirculation by CEUS in AKI is still lacking, and having insufficient observation time.

### Objectives

The present study is designed primarily to investigate the characteristics of these CEUS derived parameters in biopsy-proven AKI patients, and explore the relationship with clinical and pathological markers. The secondary aim is to investigate the 4-week, 12-week renal outcome and overall mortality, and examine the value of these CEUS derived parameters for predicting good renal function restoration of AKI. Our findings may provide evidence for early detection of microperfusion decrease in AKI patients, and beneficial to timely initiate fluid therapy for better renal restoration.

## Methods and design

### Study design

**Fig. 1:**
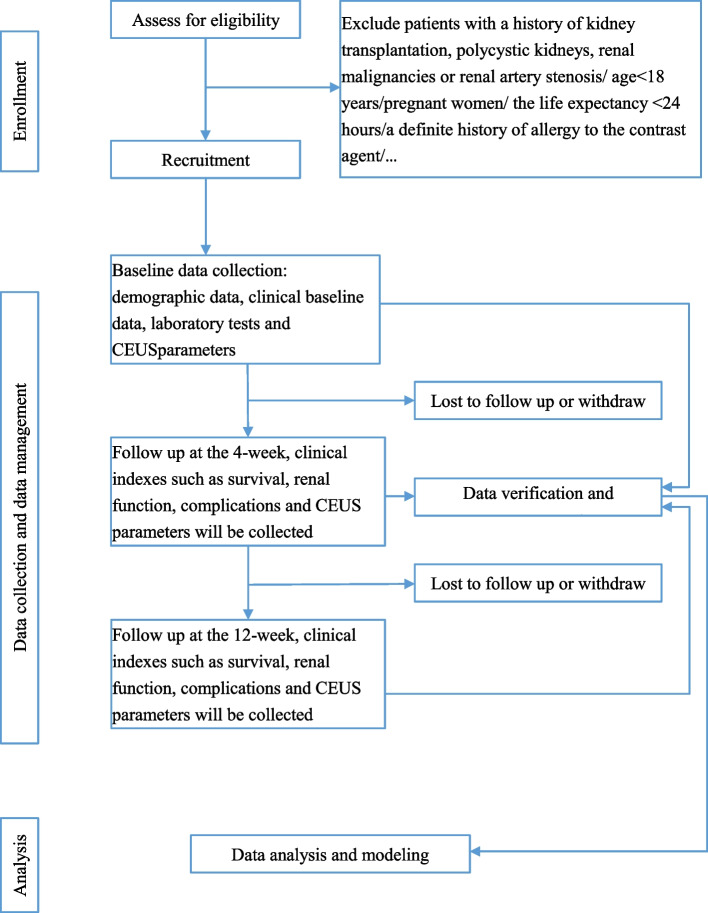
Flow diagram of our study

This study is designed as a prospective cohort study. CEUS examinations will be performed on AKI patients (study subjects) enrolled from Peking University First Hospital between September 2022 and September 2023. All enrolled patients or their guardians will sign informed consent forms. Figure 1 shows the study procedure of our study. A schematic overview of the study assessments is presented in Table 1.

**Table 1:** Overview of the study showing type of data collected for each time point

Protocol Details	Screening	Week-4	Week-12
*Informed Consent*	√	√	√
*Demographics*	√	×	×
*Clinical Information*	√	√	√
*Local lab value collection*	√	√	√
*Blood and urine Collection*	√	√	√
*CEUS parameters*	√	√	√

Organizational structure.

Our study has two functional teams: (1) clinician team of nephrology and ultrasound, responsible for designing the entire process, checking data quality and statistical analysis; and (2) supporting teams that are responsible for constructing an information system, collecting baseline information, maintenance and updates data.

Research participants.

We plan to recruit 120 in-hospital and non-ICU patients diagnosed with AKI.

The inclusion criteria are.

1. Patients clinically diagnosed with AKI following 2012 KDIGO criteria [13].
2. Patients pathologically diagnosed with tubulointerstitial nephritis will be included.
3. Complete the CEUS series examination.
4. Informed consent signed to participate in the study.

The exclusion criteria are.

1. Age < 18 years or pregnant,
2. Patients with a history of kidney transplantation,
3. Patients with polycystic kidneys or renal malignancies,
4. Renal artery stenosis > 50%,
5. The life expectancy < 24 h,
6. Definite history of allergy to the contrast agent,
7. Ultrasound images of satisfactory quality cannot be obtained,
8. Patients with hemodynamic instability, including persistent hypotension (blood pressure < 90/60 mmHg), uncontrolled cardiac insufficiency, active bleeding (such as gastrointestinal and cerebral hemorrhage, etc.) or bleeding tendency with the hemoglobin < 60 g/l and/or platelet count < 50 × 10^9^/l,
9. Patients who currently requiring mechanical ventilation, and/or stayed at the ICU,
10. Patients with a concurrent biopsy-proven severe glomerulopathy definitely contributing to AKI, such as crescentic glomerulonephritis,
11. Patients who were clinically and/or pathologically diagnosed diabetic nephropathy.

### Data collection

Demographic information, including age, sex, BMI, will be first collected. Causative factors leading to the occurrence of AKI, clinical baseline data (i.e.the underlying kidney diseases), the Acute Physiology and Chronic Health Evaluation II (APACHE II) score or SOFA score, AKI staging, average systemic blood pressure (aSBP, aDBP, MBP) will be recorded. Laboratory tests include assessment of serum creatinine, cystatin C, atrial blood natriuretic peptide, albumin concentration, 24-h urine sodium, and urine albumin to creatinine ratio. Two experienced pathologists would make a pathological diagnosis. Average scores evaluating tubulointerstitial injuries are yielded accordingly.

### Conventional B-mode ultrasonography and CEUS

Conventional B-mode ultrasonography and CEUS will be performed within 7 days post-kidney biopsy. It will be performed using a 3.5–5 MHz convex probe to evaluate the location, size, morphology, cortical thickness in the longitudinal and transverse plane. Color Doppler will be performed to assess the velocity (the peak systolic velocity and end-diastolic velocity) and resistance index of the main renal artery, segmental artery, and interlobar artery.

Based on the gray-scale ultrasound, a lower mechanical index will be applied. The patients will be placed in a lateral position, and we will choose the best longitudinal plane under grayscale ultrasound. Then enter the CEUS mode. Sonovue® (Bracco, Italy) will be chosen as the contrast agent with a bolus of 1.2–2.4 mL and administered intravenously, followed by rapid injection of 5 ml normal saline. After the infusion, we will observe contrast agents developing in the renal artery, cortex and medulla sequentially. The renal microperfusion will be observed and recorded continuously for 3 min. If the contrast agent needs to be injected again, the interval is 15 min at least. We will use Tomtec software for post-processing. The regions of interest will be outlined in the cortex and medulla of the kidney's upper, middle, and lower poles. Thus, we can obtain the relevant CEUS parameters through the fitted time-intensity curve. CEUS derived parameters include peak intensity (PI), reflecting renal micro-perfusion. Time-related indicators associated with microcirculation are rising time (RT), time to peak Intensity (TTP), mean transit time (mTT).

### Follow-up and renal outcomes

Patients will then be followed up at the 4-week and 12-week after kidney biopsy. Clinic investigation, laboratory examination, and CEUS at each follow-up will be reviewed.

The primary aim is to observe the changes of clinical and ultrasonic parameters from baseline to week 4 and week 12, including Scr, eGFR, uACR and CEUS derived parameters, the second aim is to observe the 4-week and 12-week renal outcomes. Renal outcomes will be assessed by the decrease percentage of Scr, uACR and the 4-week, 12-week eGFR level. If patients achieve a decrease percent of Scr > 50%, or an 12-week eGFR > 60 ml/min per 1.73m^2^ will be regarded as with good renal restoration.

### Primary outcome

The primary outcome is to observe the cortical and medullary perfusion changes assessed by CEUS from baseline to week 4 and week 12. These CEUS derived parameters are PI, RT, TTP and mTT.

### Secondary outcomes

• To explore the correlation of CEUS parameters with AKI severity (clinically subclassified by KDIGO stages 1–3; pathologically subclassified into high/low active and chronic injury scores).
• To identify if CEUS parameters predict prognosis of AKI (AKI reversible rate, defined as 4-week SCr decrease percent > 50%; CKD conversion rate, defined as if 12-week eGFR<60 ml/min per 1.73 m.^2^)
• To explore the relationship between CEUS parameters and renal pathology.

### Statistical analysis

All statistical analyses will be performed using SPSS software V.24.0 and MedCalc software. A P-value < 0.05 will be considered statistically significant.

For measurement data with normal distribution, mean ± standard deviation will be used for statistical description; for non-normal distribution data, the median will be used for the statistical report. For classified variables, the chi-square test will be used for statistical description. Correlation analysis will be performed to explore the relationship within clinical, pathological factors and CEUS-derived parameters. For the renal outcome of achieving eGFR ≥ 60 ml/min/1.73m^2^ at 12 weeks after kidney biopsy, we used the lassologit procedure in the stata software (version 14.2, StataCorp LLC., College Station, TX, USA.) to select potential affecting variables to be included in the logistic regression model. The odds ratios with 95% confidence interval for the selected factors will be reported. The specificity and sensitivity are yielded by a receiver operating characteristic curve analysis to examine the predictive performance of CEUS to AKI outcome.

## Discussion

AKI is a complicated clinical syndrome with high morbidity and mortality. Critically ill patients who suffered from a disorder of intra-renal microcirculation and relevant tissue hypoxia might present a sudden loss of renal function as AKI. Sepsis, cardiac dysfunction, nephrotoxic agent are common pathogenic causes leading to obvious tissue hypoperfusion, although the systemic blood pressure is normal. However, there isn’t any available method to detect the decrease of renal microcirculation sensitively. It is of value to achieve early detection because timely fluid treatment may change the therapeutic effect and poor prognosis of kidney injury. The etiology of AKI is diverse, and the pathophysiological mechanisms are complex, but changes in renal perfusion are considered important in the occurrence of AKI [14] for susceptible individuals. However, several studies in humans and animals demonstrated that AKI might develop during renal blood flow was normal or even increased. Dysfunction of the intra-renal micro-perfusion appears to be more important than reduced renal blood flow in the development and propagation of AKI [15]. In sepsis-associated AKI, sepsis can cause a profound alteration of microvascular function resulting in a heterogeneous and sluggish flow. This may lead to areas of hypoperfusion and micro-ischemia within the kidney [16]. Moreover, Lankadeva [17] et al. mentioned that renal medullary hypoxia due to redistribution of intra-renal perfusion was also considered a critical mediator of septic AKI. Ma [18] et al. also agree that renal tissue hypoperfusion and hypoxia are essential mediators of the pathogenesis of multiple forms of AKI. In addition, recent experimental studies show that in septic AKI, the medulla is more significantly damaged than the cortex, both in terms of microperfusion and hypoxia [4]. Therefore, our study plans to explore the changes of microperfusion both in the cortex and in the medulla in a prospective cohort, and evaluate long-term renal outcome.

At Present, the diagnosis of AKI is mainly based on guidelines developed by KDIGO [13]. Serum creatinine and urine volume are used as diagnostic and staging criteria. However, KDIGO's standard has some limitations. First, the evidence for using urine volume as the sole criterion for defining or staging AKI is not convincing because short-term oliguria may simply reflect insufficient volume resuscitation [19], and urine volume is not better than the serum creatinine criteria of the classification as the predictor of mortality [20]. Second, It is difficult to determine baseline creatinine and renal function in patients with AKI without baseline serum creatinine levels. Therefore, a more sensitive test is needed to detect changes in kidney function at an earlier stage of AKI.

CEUS is regarded as a promising non-invasive method able to test tissue microcirculation. We can obtain contrast-enhanced images that reflect the perfusion of normal and abnormal tissues by contrast-enhanced ultrasonography (CEUS), which is mainly based on the backscattering of gas microbubbles in the blood in the sound field. This technique has a high spatial and temporal resolution and can dynamically display renal microperfusion in real-time. Combined with various parameters of the time-intensity curve (TIC), the microperfusion of the renal cortex and medulla could be quantitatively evaluated. CEUS has several advantages. First, CEUS can display the microperfusion of renal parenchyma (cortical and medulla) in patients with AKI, making up for the deficiency that Doppler ultrasound can only provide information of blood flow in patients large vessels.

Moreover, the contrast agent micro-bubble diameter is similar to that of red blood cells, and it has good scattering characteristics. And the gas is breathed out through the lung without being filtered or secreted by the kidney, so it has no nephrotoxicity. Therefore, for patients with renal impairment, it is a more appropriate choice than CT or MRI. Some clinical and animal experimental studies have been conducted on the application of CEUS in surgery-related AKI and sepsis-related AKI. We conclude that the most significant parameters for AKI diagnosis are time-related parameters, which are manifested as the extension of perfusion time. For example, Yoon [1] et al. found that RT in the renal cortex could be used as an independent indicator to predict AKI3, related to the increased resistance of glomeruli and peritubular capillaries. Harrois^6^ et al. conducted a prospective study on 20 ICU patients with septic shock. They concluded that CEUS parameter mTT in patients with AKI (stage 2 and 3) was significantly higher than without AKI and AKI (stage 1). And compared with RI, the predictive value of mTT was higher for AKI (stages 2 and 3) within 24 h. In addition to the study on a single factor, some scholars have constructed models based on clinical and CEUS parameters. Wang [21] et al. believed that the combined use of Scr, WiR, and PI could achieve higher accuracy (AUC = 0.943) in diagnosing sepsis-associated AKI. The accuracy of diagnosing AKI in sepsis patients was significantly higher than that of a single index, with the sensitivity and specificity of 94.59% and 81.13%, respectively. All the studies mentioned above are related to the diagnosis of AKI. As for AKI prognosis, Yoon [1] et al. found that cortical RT and mTT were valuable parameters for predicting renal replacement therapy, cortical WiR and medullary PI were sensitive parameters for predicting AKI recovery, and medullary PI and AUC could predict the progression of AKI to chronic kidney disease. Though there have been some studies on applications of CEUS in AKI, further exploration is still needed in this field.

During the follow-up, our study expects to monitor microperfusion changes in the renal cortical and medullary by CEUS. Besides, we intend to collect and record clinical data, combined with pathological index and CEUS derived parameters. Thus, we can analyze if CEUS parameters could reflect renal function or assess the prognosis of patients.

Our study has some strengths. At present, there are few prospective studies on assessments of renal perfusion in patients with AKI. The major strengths of the study in relation to previous work are: The comparison with pathological information from contemporaneous renal biopsy; The longer follow-up period than in previous studies will provide important information on renal outcome following AKI. Our study also has some limitations. Firstly, as an initial study, we plan to select individuals with tubulointerstitial nephropathy, and patients with complicated glomerulopathy such as diabetic nephropathy will be excluded. Secondly, CEUS images are based on gray-scale ultrasound; the factors that affect the gray-scale ultrasound images can also affect the CEUS images. Therefore, it is necessary to perform CEUS under the condition of obtaining good gray-scale ultrasound images. Besides, the examination range of CEUS is small and cannot examine multiple lesions simultaneously; repeated injections of contrast agents are required to observe various lesions.

In a word, CEUS has been expected to provide a new method for detecting changes of microperfusion of kidneys in the early stages of AKI, which may help determine a clinical diagnosis and predict progression and prognosis of AKI. This protocol is an innovative and preliminary exploration in the field of application of CEUS in AKI. We expect to explore the changes of renal microperfusion in patients with AKI and to explore its value in predicting the severity and prognosis of AKI.

## Data Availability

The results will be published on the clinical trial website and shared with the worldwide medical community within 2 years of recruitment.

## Abbreviations

AKI: Acute kidney injury
CEUS: Contrast-enhanced ultrasonography
KDIGO: Kidney disease: Improving Global Outcomes
eGFR: Estimated glomerular filtration rate
CT: Computed tomography
MRI: Magnetic resonance imaging
rBV: Relative blood volume
RT: Rising time
mTT: Mean transit time
PI: Peak intensity
TTP: Time to peak intensity
WiR: Wash-in rate
WoR: Wash-out rate
AUC: Receiver operating characteristics curve
CKD: Chronic kidney disease
DKD: Diabetes kidney disease
ADHF: Acute decompensated heart failure
APACHE II: Acute physiology and chronic health evaluation II
SOFA: Sepsis-related organ failure assessment
aSBP: Average systemic blood pressure
aDBP: Artery diastolic blood pressure
MBP: Mean arterial pressure
TIC: Time-intensity curve
ICU: Intensive care unit
